# Pericytes Stimulate Oligodendrocyte Progenitor Cell Differentiation during CNS Remyelination

**DOI:** 10.1016/j.celrep.2017.08.007

**Published:** 2017-08-22

**Authors:** Alerie Guzman De La Fuente, Simona Lange, Maria Elena Silva, Ginez A. Gonzalez, Herbert Tempfer, Peter van Wijngaarden, Chao Zhao, Ludovica Di Canio, Andrea Trost, Lara Bieler, Pia Zaunmair, Peter Rotheneichner, Anna O’Sullivan, Sebastien Couillard-Despres, Oihana Errea, Maarja A. Mäe, Johanna Andrae, Liqun He, Annika Keller, Luis F. Bátiz, Christer Betsholtz, Ludwig Aigner, Robin J.M. Franklin, Francisco J. Rivera

**Affiliations:** 1 Wellcome Trust and MRC Cambridge Stem Cell Institute, University of Cambridge, Cambridge CB20AH, UK; 2 Institute of Molecular Regenerative Medicine, Paracelsus Medical University Salzburg, 5020 Salzburg, Austria; 3 Spinal Cord Injury and Tissue Regeneration Center Salzburg (SCI-TReCS), Paracelsus Medical University Salzburg, 5020 Salzburg, Austria; 4 Laboratory of Stem Cells and Neuroregeneration, Institute of Anatomy, Histology and Pathology, Faculty of Medicine, Universidad Austral de Chile, Valdivia, Chile; 5 Center for Interdisciplinary Studies on the Nervous System (CISNe), Universidad Austral de Chile, Valdivia, Chile; 6 Institute of Pharmacy, Faculty of Sciences, Universidad Austral de Chile, Valdivia, Chile; 7 Institute for Tendon and Bone Regeneration, Paracelsus Medical University Salzburg, 5020 Salzburg, Austria; 8 Austrian Cluster for Tissue Regeneration, Vienna, Austria; 9 Centre for Eye Research Australia, Royal Victorian Eye and Ear Hospital, Ophthalmology, Department of Surgery, University of Melbourne, Australia; 10 Ophthalmology/Optometry and Research Program for Experimental Ophthalmology, Paracelsus Medical University Salzburg, 5020 Salzburg, Austria; 11 Institute of Experimental Neuroregeneration, Paracelsus Medical University Salzburg, 5020 Salzburg, Austria; 12 Department of Immunology, Genetics and Pathology, Rudbeck Laboratory, Uppsala University, 751 85 Uppsala, Sweden; 13 Department of Neurosurgery, Tianjin Medical University General Hospital, Tianjin Neurological Institute, Key Laboratory of Post-Neuroinjury Neuro-Repair and Regeneration in Central Nervous System, Ministry of Education and Tianjin City, Tianjin 300052, China; 14 Division of Neurosurgery, Zürich University Hospital, Zürich University, 8091 Zürich, Switzerland; 15 Centro de Investigación Biomédica (CIB), Facultad de Medicina, Universidad de los Andes, Santiago, Chile; 16 Integrated Cardio Metabolic Center (ICMC), Karolinska Institutet Novum, 141 57 Huddinge, Sweden

**Keywords:** neurovascular niche, pericytes, remyelination, oligodendrocyte progenitor cell, Lama2

## Abstract

The role of the neurovascular niche in CNS myelin regeneration is incompletely understood. Here, we show that, upon demyelination, CNS-resident pericytes (PCs) proliferate, and parenchymal non-vessel-associated PC-like cells (PLCs) rapidly develop. During remyelination, mature oligodendrocytes were found in close proximity to PCs. In *Pdgfb*^ret/ret^ mice, which have reduced PC numbers, oligodendrocyte progenitor cell (OPC) differentiation was delayed, although remyelination proceeded to completion. PC-conditioned medium accelerated and enhanced OPC differentiation in vitro and increased the rate of remyelination in an ex vivo cerebellar slice model of demyelination. We identified Lama2 as a PC-derived factor that promotes OPC differentiation. Thus, the functional role of PCs is not restricted to vascular homeostasis but includes the modulation of adult CNS progenitor cells involved in regeneration.

## Highlights

• CNS-resident PCs react to demyelination and are found close to differentiating OPCs
• PC-deficient mice show delayed OPC differentiation during CNS remyelination
• PC-conditioned medium accelerates OPC differentiation and enhances remyelination
• PC-derived LAMA2 induces OPC differentiation

## Graphical Abstract

**Figure:**
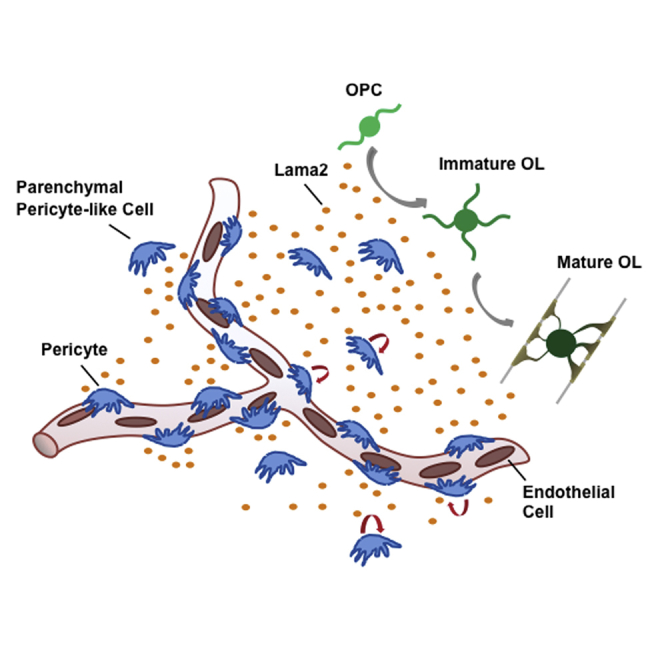

## Teaser

Following toxin-induced demyelination in PC-deficient mice and using a number of in vitro approaches, Guzman de la Fuente et al. show that CNS pericytes (PCs) respond to demyelination and, through Lama2 secretion, stimulate oligodendrocyte progenitor cell differentiation during remyelination. These findings extend PC function beyond vascular homeostasis toward regeneration.

## Introduction

Although the adult mammalian CNS has a limited capacity for regeneration, lost oligodendrocytes and the myelin they produce are restored during remyelination. In response to demyelination, oligodendrocyte progenitor cells (OPCs) proliferate and migrate to the lesion, where they differentiate into remyelinating oligodendrocytes (Franklin and Ffrench-Constant, 2008, Zawadzka et al., 2010). Remyelination restores saltatory conduction and ensures axonal survival, and its impairment renders demyelinated axons vulnerable to irreversible degeneration (Franklin et al., 2012). Thus, promoting remyelination is a major therapeutic objective in chronic demyelinating diseases such as multiple sclerosis, where remyelination becomes increasingly inefficient with disease progression (Goldschmidt et al., 2009).

Myelination during development is strictly coupled to angiogenesis (Yuen et al., 2014), where OPCs use the vasculature as scaffolds for migration (Tsai et al., 2016). Similarly, regeneration in the CNS is coupled to the vascular niche (Manavski et al., 2014). The CNS vascular niche consists of endothelial cells (ECs), vasculature-associated cells such as perivascular cells, astrocytes, neurons, OPCs, molecules derived from these cells, and blood-derived elements. Pericytes (PCs), which are located at the abluminal surface of capillaries and embedded within the basement membrane, regulate angiogenesis and neovascularization and control microvascular blood flow (Armulik et al., 2010). The CNS contains the highest PC density in the body, ensuring proper blood-brain barrier (BBB) structure and function (Armulik et al., 2010, Winkler et al., 2011). CNS-resident PCs also share features with mesenchymal stem cells (MSCs), expressing similar genes and differentiating into cells of the mesenchymal lineage in vitro (Crisan et al., 2008). MSCs secrete a plethora of paracrine signaling molecules (Chen et al., 2009), including factors that promote oligodendrocyte differentiation (Jadasz et al., 2013, Rivera et al., 2006). We hypothesized that PCs act in a similar fashion (Lange et al., 2013). This assumption is supported by the close physical proximity between PCs and OPCs in healthy cerebral white matter (Maki et al., 2015) and by the ability of PC-derived transforming growth factor β 1 to regulate OPC migration during cortical development (Choe et al., 2014). Here we analyzed PCs, their response to a demyelinating lesion, and their functional role in oligodendrocyte differentiation during remyelination. In vivo relevance was studied through a genetic mouse model with reduced pericyte coverage. Moreover, molecular and cellular analysis identified the PC-derived factor modulating OPC differentiation.

## Results

### Pericytes Respond to CNS Demyelination

**Figure 1:**
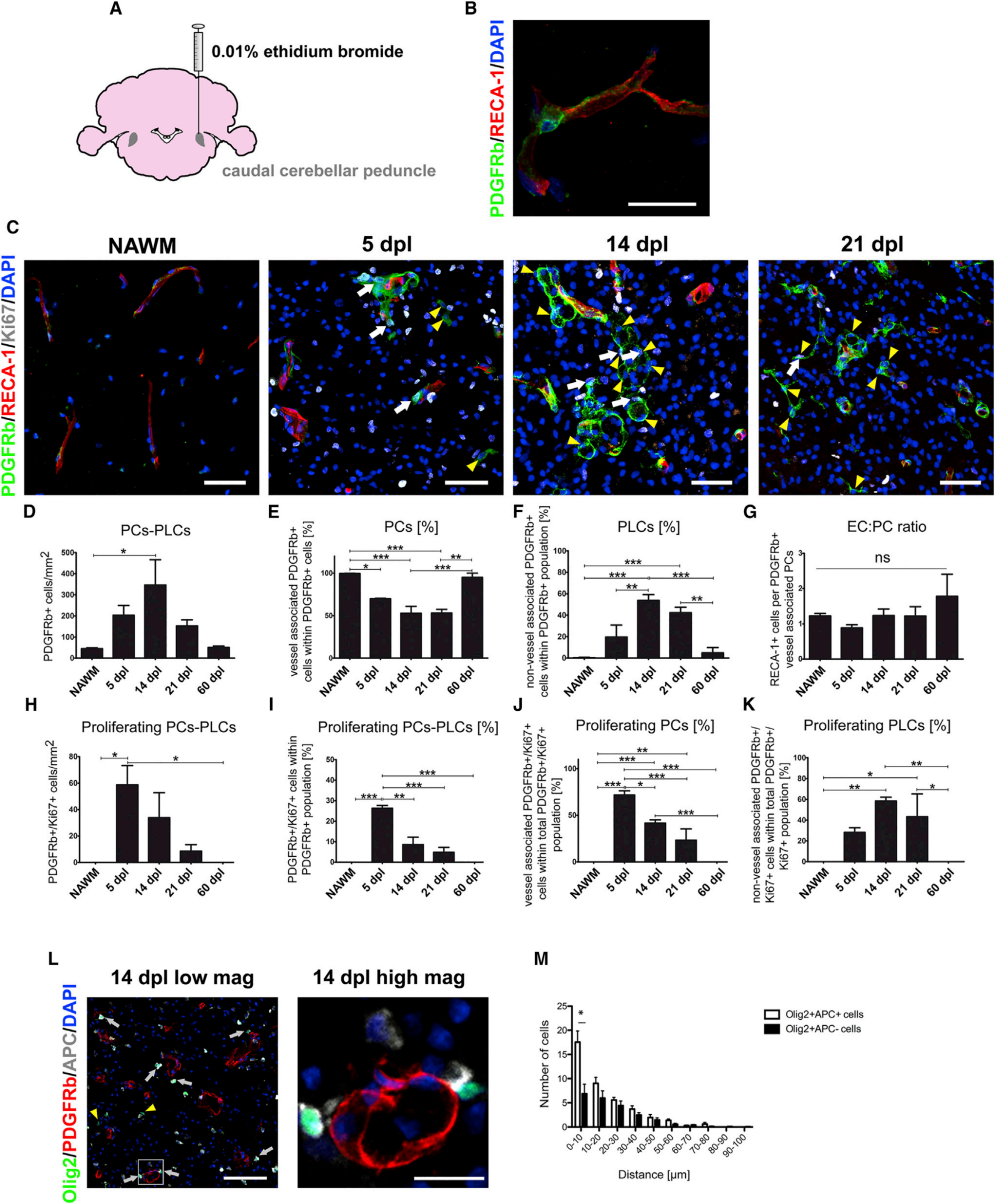
Pericytes React to Demyelination (A) Ethidium bromide-induced demyelinated lesion in the CCP of adult rats. (B) PDGFRb expressing PC and RECA-1+ ECs in NAWM. (C) Proliferating PDGFRb+ cells and ECs (PDGFRb/RECA-1/Ki67) within lesions at 5, 14, and 21 dpl. White arrows indicate proliferating PCs and yellow arrowheads PLCs. (D–K) Quantitative analyses of different PC parameters in response to demyelination. (L) Immunohistochemistry showing the distance of OPCs (Olig2+/APC−), mature oligodendrocytes (Olig2+/APC+), and PDGFRb+ at 14 dpl; yellow arrowheads indicate OPCs and white arrows mature oligodendrocytes. The high-magnification image shows mature oligodendrocytes close to PDGFRb+ cells. (M) The graph displays the distance distribution of OPCs and mature oligodendrocytes from PDGFRb + cells. (C–M) 3 or more animals were analyzed for each time point. (L and M) 150 or more distance measurements were performed from oligodendrocytes to PDGFRb+ cells per animal. Means ± SEM are shown. Data were analyzed by one-way ANOVA followed by Tukey’s post hoc test, Student’s t test, or chi-square test. ^∗^p < 0.05, ^∗∗^p < 0.01, ^∗∗∗^p < 0.001. Scale bars, (B) 20 μm; (C) 50 μm; (D) 50 μm, high-magnification 10 μm; (E) 50 μm, high magnification 10 μm.

We first analyzed the proliferative response and localization of ECs and PCs following induction of white matter demyelination upon injection of ethidium bromide into the caudal cerebellar peduncles (CCPs) of adult rats (Figure 1A). In this model, demyelination occurs within 24 hr of injection and induces OPC proliferation and migration, which peaks at 5 days post-lesion induction (dpl), followed by OPC differentiation, which is ongoing at 14 dpl. Remyelination is completed by 21 dpl. There was a significant increase in proliferating (Ki67-positive) rat endothelial cell antigen 1 (RECA-1)-expressing ECs and a robust proliferative response of platelet-derived growth factor receptor beta (PDGFRb)-expressing PCs within the demyelinated lesion at 5 dpl (Figures S1C and S1D), although the vessel area was not altered between 5 and 21 dpl (Figures S1A and S1B). In the normal-appearing white matter (NAWM), PCs are restricted to the perivascular region (Figure 1B). The total number of PDGFRb+ cells within the lesion was significantly increased at 14 dpl compared with NAWM (Figures 1C and 1D). This was accompanied by the appearance of PDGFRb+ cells away from blood vessels, which, because we cannot be certain of their origin, we defined as pericyte-like cells (PLCs) to distinguish them from PCs close to blood vessels (Figure 1C). Within the whole PDGFRb+ population (i.e., PCs and PLCs), the proportion of PCs declined during remyelination (Figure 1E), whereas the percentage of parenchymal PLCs increased and was highest at 14 dpl (Figure 1F). Consistent with the unaltered density of ECs and PCs during remyelination (Figures S1E and S1F), the EC:PC ratio, crucial for BBB stability (Armulik et al., 2010), remained the same (Figure 1G). At 5 dpl, there were numerous Ki67+ PDGFRb+ cells in the lesion that peaked at 14 dpl but declined thereafter and were absent at 60 dpl (Figures 1H–1K). By 60 dpl, when the lesion is fully remyelinated, PDGFRb+ cell numbers and localization were similar to that in NAWM, where only PCs and no PLCs were found (Figures 1C–1G). Staining for desmin, another PC marker, supported these findings (Figure S4A).

We hypothesized that, if PCs and/or PLCs were involved in the differentiation of OPCs into remyelinating oligodendrocytes, then the differentiated cells would tend to be located in close proximity to PDGFRb+ cells. We therefore assessed the distance between Olig2+/adenomatous polyposis coli (APC)− OPCs and Olig2+/APC+ oligodendrocytes to the nearest PDGFRb+ cell at 14 dpl. We found that oligodendrocytes were preferentially located close (i.e., within 10 μm) to PCs, whereas OPCs showed no preferential clustering close to these cells; neither was there clustering of either OPCs or oligodendrocytes close to PLCs (Figures 1L and 1M; Figures S1G and S1H). These results suggest that PCs and PLCs may have differential roles in modulating OPC function during remyelination.

### Impaired Oligodendrocyte Differentiation during Remyelination in PC-Deficient Mice

**Figure 2:**
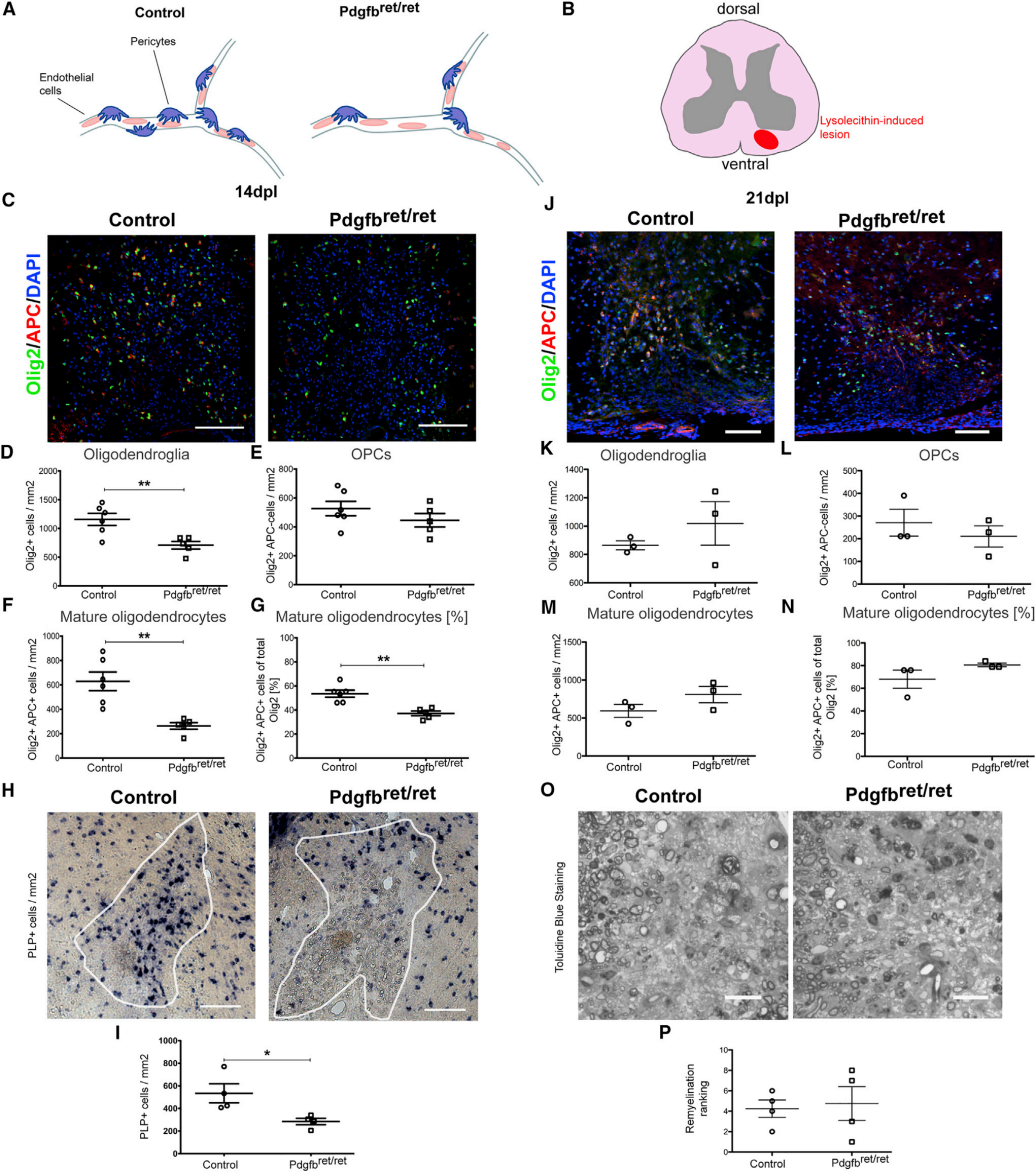
OPC Differentiation Is affected during Remyelination in *Pdgfb*^ret/ret^ Mice (A and B) Illustrations of decreased PC coverage in *Pdgfb*^ret/ret^ mice (A) and of lysolecithin-induced demyelination (B). (C and J) Mature oligodendrocytes (Olig2+/APC+) and OPCs (Olig2+/APC−) in lesions of control and *Pdgfb*^ret/ret^ mice at 14 dpl (C) and 21dpl (J). (D–G and K–N) Quantitative analyses of oligodendroglia at 14 dpl (D–G) and 21 dpl (K–N). (H and I) In situ hybridization and quantification for PLP of control and *Pdg*fb^ret/ret^ mice at 14 dpl. (O and P) Toluidine blue staining of remyelination in control and *Pdgfb*^ret/ret^ mice at 21 dpl (O) and its quantification by relative ranking analysis (P). Means ± SEM are shown. Data were analyzed by Student’s t test or Mann-Whitney *U* test. ^∗^p < 0.05, ^∗∗^p < 0.01, ^∗∗∗^p < 0.001. Scale bars, 100 μm.

To determine whether CNS-resident PCs influence remyelination, we used *Pdgfb*^ret/ret^ mice, in which a mutation in the retention motif of the *Pdgfb* gene causes a 75%–85% reduction in capillary PC coverage compared with heterozygous littermates (Figure 2A; Armulik et al., 2010, Lindblom et al., 2003). PDGFRb is still expressed in these mice and can still be used as a marker for residual pericytes.

We first examined myelination in *Pdgfb*^ret/ret^ mice and found that, on post-natal day 12 (P12), the density of mature oligodendrocytes (Olig2+/APC+) was lower and the density of OPCs (Olig2+/APC−) higher than in control mice. However, on P17, no differences were observed in OPC differentiation and myelination (Figure S2).

Following lysolecithin-induced demyelination in the ventral spinal cord white matter (WM), the number of PDGFRb+ increased substantially in both *Pdgfb*^ret/ret^ and control mice. The number of PDGFRb+ cells in *Pdgfb*^ret/ret^ mice at 5 dpl was less than in control mice, and there were fewer PLCs. However, by 14 dpl, the density of PDGFRb+ cells was the same in both groups because of a higher proliferative response of these cells in the *Pdgfb*^ret/ret^ mice (Figures S3A–S3F). No differences were detected in the astrocyte or in the macrophage/microglial response between *Pdgfb*^ret/ret^ mice and control mice (Figures S3G–S3I). Microglia/macrophages showed the same pro-inflammatory and anti-inflammatory cell profile distribution in both groups (Figures S3J–S3L), and no differences were detected in BBB stability in the lesion area (Figures S3M–S3Q).

The total number of Olig2+ cells within the lesion was reduced in *Pdgfb*^ret/ret^ mice compared with controls at 14 dpl. There was no significant difference in the number of Olig2+/APC− OPCs between *Pdgfb*^ret/ret^ and control mice (Figures 2C and 2E). However, the number of Olig2+/APC+ mature oligodendrocytes was significantly reduced in *Pdgfb*^ret/ret^mice (Figures 2C, 2F, and 2G). In situ hybridization for proteolipid protein (PLP), a major myelin component, generated consistent results (Figures 2H and 2I). However, the densities of Olig2+ cells and Olig2+APC+ oligodendrocytes were the same in both groups by 21 dpl (Figures 2J–2N). No differences were detected in the extent of remyelination, as assessed by ranking analysis of toluidine blue-stained resin sections in which demyelinated and remyelinated axons were discernable (Figures 2O and 2P).

These data indicate that *Pdgfb*^ret/ret^ mice show a delay in OPC differentiation because of the decreased numbers of PDGFRb+ cells at the earlier stages following demyelination. However, because the PDGFRb+ cell numbers normalize by 14 dpl, this delay does not translate into a decrease in remyelination.

### PCs Promote OPC Differentiation and Enhance Remyelination Ex Vivo

**Figure 3:**
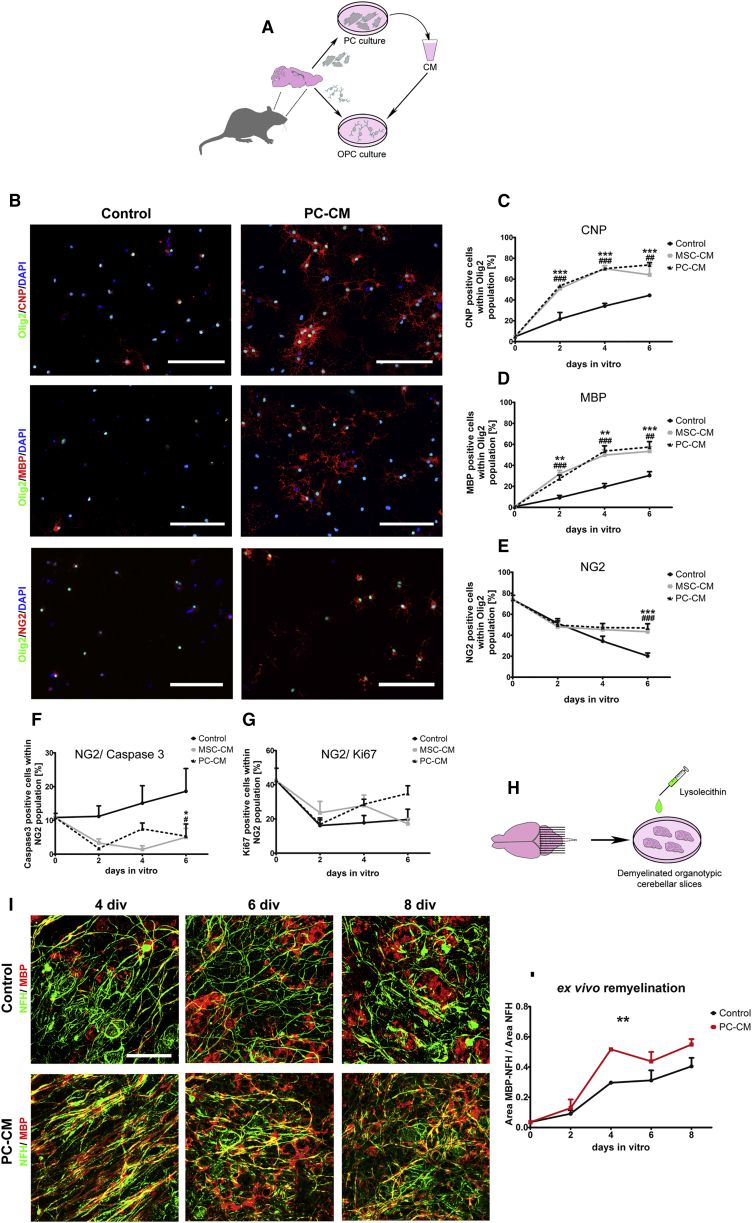
PCs Promote OPC Differentiation and Accelerate Remyelination in Demyelinated Organotypic Slices (A) Schematic of the experimental design. (B–E) Fluorescence images (B) displaying CNP+/Olig2+, MBP+/Olig2+, and NG2+/Olig2+ oligodendrocytes at 4 DIV and their respective graphs (C–E). (F and G) PC-CM increases the generation of oligodendrocytes from OPCs. Graphs display (F) the OPC survival rate (NG2+/Caspase+) and (G) the OPC proliferation rate (NG2+/Ki67+) under different conditions. (H) Illustration of lysolecithin-induced demyelination of slice cultures. (I) Images showing axons (NFH+) and myelin (MBP+) of remyelinating cerebellar slices exposed to control medium versus PC-CM at 4, 6, and 8 days post lysolecithin treatment. (J) Quantification of the MBP/NFH ratio shows that PC-CM increases the remyelination rate. 3 or more independent OPC and slice preparations were used. Means ± SEM are shown. Data were analyzed by two-way ANOVA followed by Bonferroni’s post hoc test. ^∗^p < 0.05, ^∗∗^p < 0.01, ^∗∗∗^p < 0.001. ^∗^ indicates statistical difference for PC-CM and # for MSC-CM. Scale bars, (B) 100 μm and (I) 50 μm.

We next examined how PCs might directly influence OPC function. We therefore exposed cultured OPCs to PC-conditioned medium (PC-CM) and evaluated the rate of differentiation. MSC-conditioned medium (MSC-CM), previously identified as a strong inducer of OPC differentiation (Jadasz et al., 2013), was used as a positive control. Primary PCs exhibiting MSCs features, including mesenchymal differentiation potential, were isolated from adult rat brains (Figure 3A; Figures S4B–S4D). Upon incubation with PC-CM, a higher proportion of OPCs had acquired 2',3′-cyclic-nucleotide 3′-phosphodiesterase (CNP) and myelin basic protein (MBP) expression at 2 days in vitro (DIV) compared with those exposed to non-conditioned control medium (Figures 3B–3D). We also found a significantly higher proportion of NG2+/Olig2+ OPCs in the PC-CM-treated cultures (Figures 3B and 3E). This effect was likely due to an anti-apoptotic effect of the PC-CM because PC-CM significantly decreased the proportion of caspase-3-positive OPCs compared with control conditions (Figure 3F). Proliferation, assessed by Ki67 staining, was not affected by PC-CM (Figure 3G).

We next examined whether PCs can enhance remyelination. We used postnatal rat-derived cerebellar organotypic slice cultures as an ex vivo model of remyelination (Yuen et al., 2013, Zhang et al., 2011). In this model, P10 rat cerebellar slices were demyelinated using lysolecithin (Birgbauer et al., 2004) and then exposed to PC-CM or control medium (Figure 3H). Remyelination was analyzed at 0, 2, 4, 6, and 8 days after lysolecithin treatment by immunohistochemical staining for MBP and neurofilament heavy chain (NFH). PC-CM significantly enhanced the remyelination rate after lysolecithin-induced demyelination (Figures 3I and 3J).

### PC-Induced OPC Differentiation Is Mediated through Lama2

**Figure 4:**
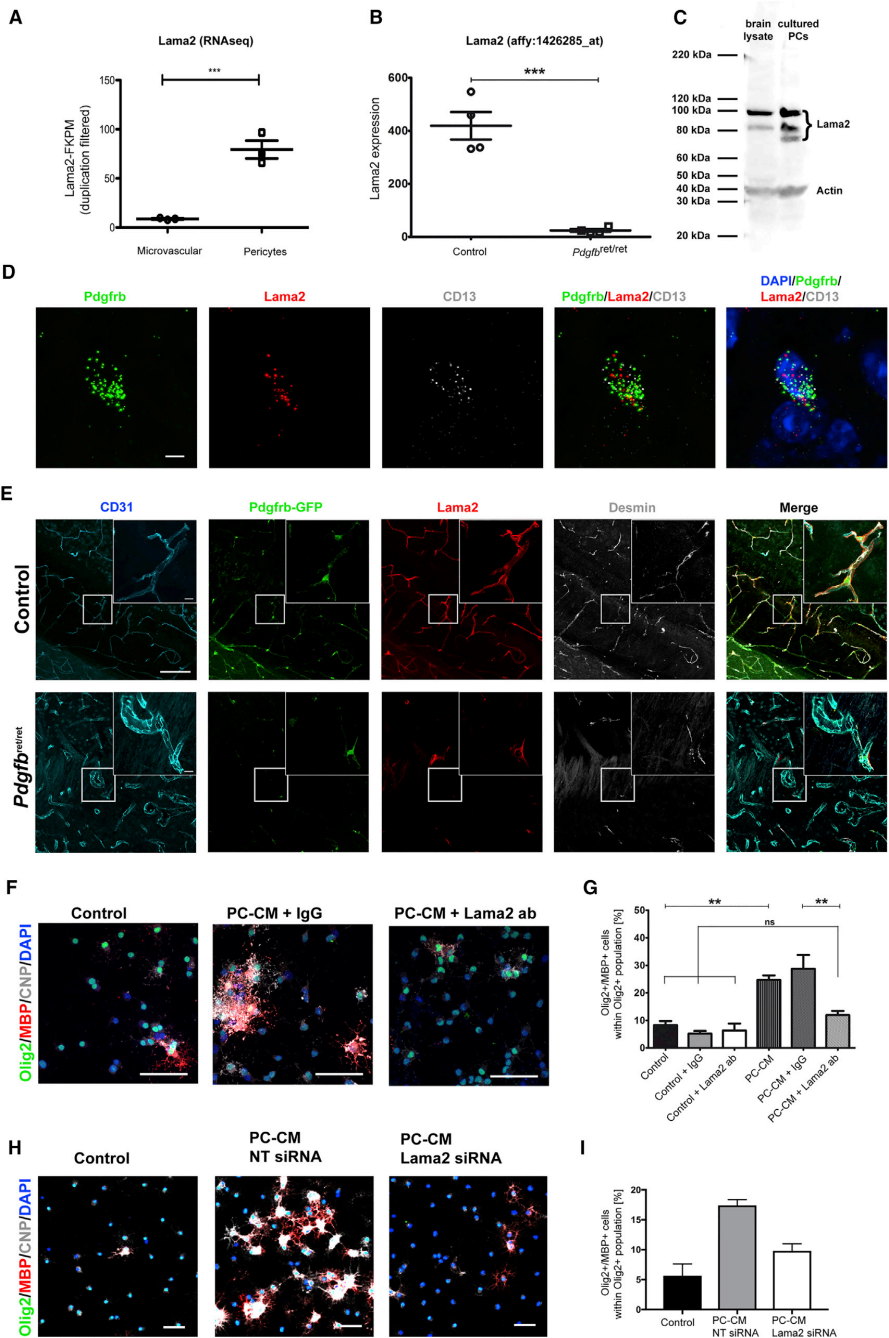
PC-Derived Lama2 Promotes OPC Differentiation and Is Decreased in *Pdgfb*^ret/ret^ Mice (A) RNA-seq data showing the expression of Lama2 in the microvasculature (mainly consisting of a majority of ECs with very low PC contribution) versus sorted PCs (data extracted from He et al., 2016). (B) Downregulation of Lama2 in microvessels of *Pdgfb*^ret/ret^ mice (Affymetrix array data extracted from He et al., 2016). (C) Western blot image indicating Lama2 expressed by PCs in vivo (brain) and in vitro. (D) RNA in situ hybridization images showing PC-specific expression of PDGFRb, CD13, and Lama2. (E) Confocal images of CD31/PDGFRb-GFP/Lama2/desmin of *Pdgfb*^ret/+^ × *Pdgfrb*-EGFP mice compared with *Pdgfb*^ret/ret^ × *Pdgfrb*-EGFP indicate PC-specific expression of Lama2 and show a decline of PCs and Lama2 in *Pdg*fb^ret/ret^ mice. Boxed areas represent high-magnification images. (F) Fluorescence images of oligodendrocytes (Olig2+/MBP+) 2 DIV with control medium, PC-CM with IgG, and PC-CM with anti-Lama2-blocking antibody. (G) Graph displaying the percentage of Olig2+/MBP+ cells. (H) Fluorescence images of OPCs in control medium, PC- CM from non-targeting siRNA, and PC Lama2 siRNA-treated-CM. (I) Graph displaying the Olig2+/MBP+ cell population in the presence of the different siRNA-treated PC-CMs. n = 4 mice per group, and 3 or more independent cell preparations were used, except for siRNA experiments, where n = 2. Means ± SEM are shown. Data were analyzed by Student’s t test or one-way ANOVA followed by Tukey’s post hoc test. ^∗^p < 0.05, ^∗∗^p < 0.01, ^∗∗∗^p < 0.001. Scale bars, (C) 5 μm; (D) 100 μm, high-magnification 10 μm; (E) and (G), 50 μm.

We have previously shown that Lama2 mRNA expression is decreased in the microvessels of *Pdgfb*^ret/ret^ mice compared with control mice (Armulik et al., 2010; Figure 4B). The *Lama2* gene product is highly expressed during remyelination (Zhao et al., 2009) and has been shown to modulate OPC function (Relucio et al., 2012). Given that the main source of Lama2 within the microvessels is PDGFRb+ cells, as shown by RNA sequencing (RNA-seq) (He et al., 2016; Figure 4A), we investigated whether Lama2 is a mediator of the PC-induced effect on OPC differentiation. RNA in situ hybridization of healthy brain sections revealed a specific expression of Lama2 in cells expressing the PC markers PDGFRb and CD13 (Figure 4D). In control mice, Lama2 was mainly detected around cell bodies and processes of PCs, whereas *Pdgfb*^ret/ret^ mice had less Lama2 protein expression that, when present, was always associated with residual PCs (Figure 4E). Cultured PCs also expressed Lama2 (Figure 4C). Pre-incubation of PC-CM with an anti-Lama2 antibody resulted in a significant decrease in the Olig2+/CNP+ and Olig2+/MBP+ populations compared with PC-CM pre-incubated with a general immunoglobulin G (IgG), reducing OPC differentiation to a level indistinguishable from control conditions (Figures 4F and 4G). These results were replicated in OPCs exposed to PC-CM harvested from PCs treated with either non-targeting or Lama2 small interfering RNA (siRNA) for 48 hr (Figures 4H and 4I). Taken together, these data identify Lama2 as a PC-derived molecule that promotes OPC differentiation.

## Discussion

This work reveals an unidentified role of PCs in CNS regeneration. We show that PCs in the vessel walls respond to demyelination by proliferation. We describe a parenchymal cell population expressing PC markers such as PDGFRb and desmin that appears soon after the induction of demyelination. Although these cells express PC markers, we cannot be certain of their origin, and, therefore, we refer to them as PLCs. Upon demyelination, both PCs and PLCs have a high proliferative response; however, mature oligodendrocytes are preferentially located in higher frequency close to PCs. These data suggest that, even though both populations respond to demyelination, they may have differential roles in their interaction with OPCs during remyelination, which will require further investigation. In mice with a reduced PC density, OPC differentiation is delayed in the demyelinated lesion, resulting in fewer mature oligodendrocytes at 14 dpl. Moreover, exposure to PC-CM accelerated OPC differentiation and enhanced ex vivo remyelination. We identified Lama2 as a key factor responsible for the effect of PCs in promoting OPC differentiation.

In humans, loss of *LAMA2* gene expression causes WM abnormalities, manifested at an early postnatal age, when developmental myelination occurs (Allamand and Guicheney, 2002). Similarly, a mouse model for Lama2 deficiency (*Lama2*^*−/−*^) shows developmental delays in oligodendrocyte differentiation and the accumulation of OPCs, leading to defects in myelination in the corpus callosum (Chun et al., 2003, Relucio et al., 2012). Lama2 regulates OPC function through its interaction with dystroglycan (Leiton et al., 2015). In the CNS, Lama2 is expressed by PCs and upregulated during OPC differentiation (14 dpl) in a toxin-induced demyelination model (Zhao et al., 2009). Lama2 seems to be relevant for PC function because *Lama2*^*−/−*^ mice have decreased microvascular PC coverage associated with BBB defects (Menezes et al., 2014). Our data suggest that PC-derived Lama2 has a key role in promoting OPC differentiation because the PC-CM effect in OPCs was inhibited when exposed to an anti-Lama2 antibody or when PCs were treated with Lama2 siRNA. Consistent with this, we observed that OPC differentiation was delayed in adult *Pdgfb*^ret/ret^ mice after lysolecithin-induced demyelination. These findings indicate that PCs provide an appropriate milieu for differentiating OPCs through Lama2.

This study indicates that PC functions are not restricted to vascular homeostasis but, rather, extend to CNS regeneration. This is supported by studies illustrating that PCs respond with high proliferation to acute lesions such as stroke or spinal cord injury (SCI), where they modulate inflammation or the formation of fibrotic scar tissue (Göritz et al., 2011, Özen et al., 2014). Here we show that, besides stabilizing CNS vasculature and regulating EC function, pericytes also have a high proliferative response following CNS demyelination and directly influence CNS-resident progenitor cell differentiation during remyelination, most likely by secretion of Lama2.

## Experimental Procedures

### Animal Work

Animal experiments within this research have been regulated under the Animals (Scientific Procedures) Act 1986 Amendment Regulations 2012 following ethical review by the University of Cambridge Animal Welfare and Ethical Review Body (AWERB) in accordance with United Kingdom Home Office regulations (Project License 70/7715) and in accordance with Austrian laws on animal experimentation and were approved by Austrian regulatory authorities (Permit No. BMWF-66.012/0001-II/3b/2014; license codes BMBF-66-012/0037-WF/V/3b/2014 and BMWF-66.012/0032-WF/V/3b/2015). During this study, 2- to 3-month-old Sprague Dawley rats and 12-week-old *Pdgfb*ret/ret mice (hetero- and homozygous), which were previously described by Armulik et al. (2010) and Lindblom et al. (2003), were used for toxin-induced demyelination.

### Cell Culture

Preparation of CNS pericytes was performed following the Dore-Duffy protocol (Dore-Duffy et al., 2006) with modifications. Rat bone marrow-derived MSCs were prepared as described previously by Rivera et al. (2006). OPCs were prepared from neonatal P0–P2 Sprague-Dawley rats, cortices and hippocampi were digested with papain solution, and dissociated cells were seeded into poly-D-lysine-coated T75 flasks. Mixed glial cultures were kept for 11 DIV in medium with DMEM (Gibco) and 10% fetal bovine serum (Biosera). OPCs were isolated as described previously (McCarthy and de Vellis, 1980).

### Organotypic Cerebellar Slices

Remyelination of rat cerebellar slices was prepared as described previously (Birgbauer et al., 2004). After 7 DIV, the medium was replaced with organotypic slice medium containing 0.5 mg/mL lysolecithin for 16 hr. After 16 hr, the medium was replaced with PC-conditioned organotypic slice medium and non-conditioned control medium.

### Statistical Analyses

Graphs show mean values ± SEM, and statistical analysis were performed using GraphPad Prism 5.0 (GraphPad) and SPSS 20 (IBM). Parametric one-way ANOVA, Tukey post hoc analyses, Student’s t test, or Mann-Whitney *U* tests (when not normally distributed) were used when comparing one parameter. For statistical analysis with two parameters, such as time course experiments with different treatments, two-way ANOVA with Bonferroni post hoc was used. For distance frequency distribution analysis, chi-square test was used (Figure 1M; Figures S1G and S1H). All experiments were performed as indicated by n in the figure legends. Significance was as follows: p^∗^ < 0.05, p^∗∗^ < 0.01, and p^∗∗∗^ < 0.001.

For further details, see the Supplemental Experimental Procedures.

## Author Contributions

S.L. and F.J.R. conceived the project. A.G.D.L.F., S.L., L.A., R.J.M.F., and F.J.R. designed the study. S.L., A.G.D.L.F., L.F.B., C.B., L.A., R.J.M.F., and F.J.R. wrote and edited the manuscript. S.L. and A.G.D.L.F. designed the figures. A.G.D.L.F., S.L., H.T., C.Z., A.K., G.A.G., L.D.C., M.A.M., J.A., C.B., R.J.M.F., L.A., and F.J.R. planned the experiments. A.G.D.L.F., S.L., M.E.S., P.v.W., P.R., O.E., C.Z., G.A.G., A.K., M.A.M., J.A., and L.D.C. conducted the experiments. A.G.D.L.F., S.L., M.E.S., H.T., A.T., P.R., A.O., L.B., A.K., M.A.M., J.A., and F.J.R. collected data. A.G.D.L.F., S.L., M.E.S., G.A.G., L.D.C., C.Z., P.R., A.T., L.H., A.K., M.A.M., and J.A. analyzed data. A.G.D.L.F., S.L., P.R., G.A.G., A.K., M.A.M., J.A., C.B., R.J.M.F., L.A., and F.J.R. interpreted data. H.T., A.T., P.R., A.O., P.Z., S.C.D., and L.B. assisted technically. R.J.M.F., L.A., and F.J.R. supervised the project. C.B., L.A., R.J.M.F., and F.J.R. supported this study financially.

## Supplemental Information

Document S1. Supplemental Experimental Procedures and Figures S1–S4

Document S2. Article plus Supplemental Information
