# Supplementary material for: Pericytes Stimulate Oligodendrocyte Progenitor Cell Differentiation during CNS Remyelination
Source: Cell Rep. 2017 Aug 22;20(8):1755–64. doi: 10.1016/j.celrep.2017.08.007 (PMC5574064; doi:10.1016/j.celrep.2017.08.007)
Supplement: Document S1. Supplemental Experimental Procedures and Figures S1–S4 [file mmc1.pdf]

Cell Reports, Volume *20*

Supplemental Information

Pericytes Stimulate Oligodendrocyte Progenitor Cell Differentiation during CNS Remyelination

Alerie Guzman De La Fuente, Simona Lange, Maria Elena Silva, Ginez A. Gonzalez, Herbert Tempfer, Peter van Wijngaarden, Chao Zhao, Ludovica Di Canio, Andrea Trost, Lara Bieler, Pia Zaunmair, Peter Rotheneichner, Anna O'Sullivan, Sebastien Couillard-Despres, Oihana Errea, Maarja A. Mäe, Johanna Andrae, Liqun He, Annika Keller, Luis F. Bátiz, Christer Betsholtz, Ludwig Aigner, Robin J.M. Franklin, and Francisco J. Rivera

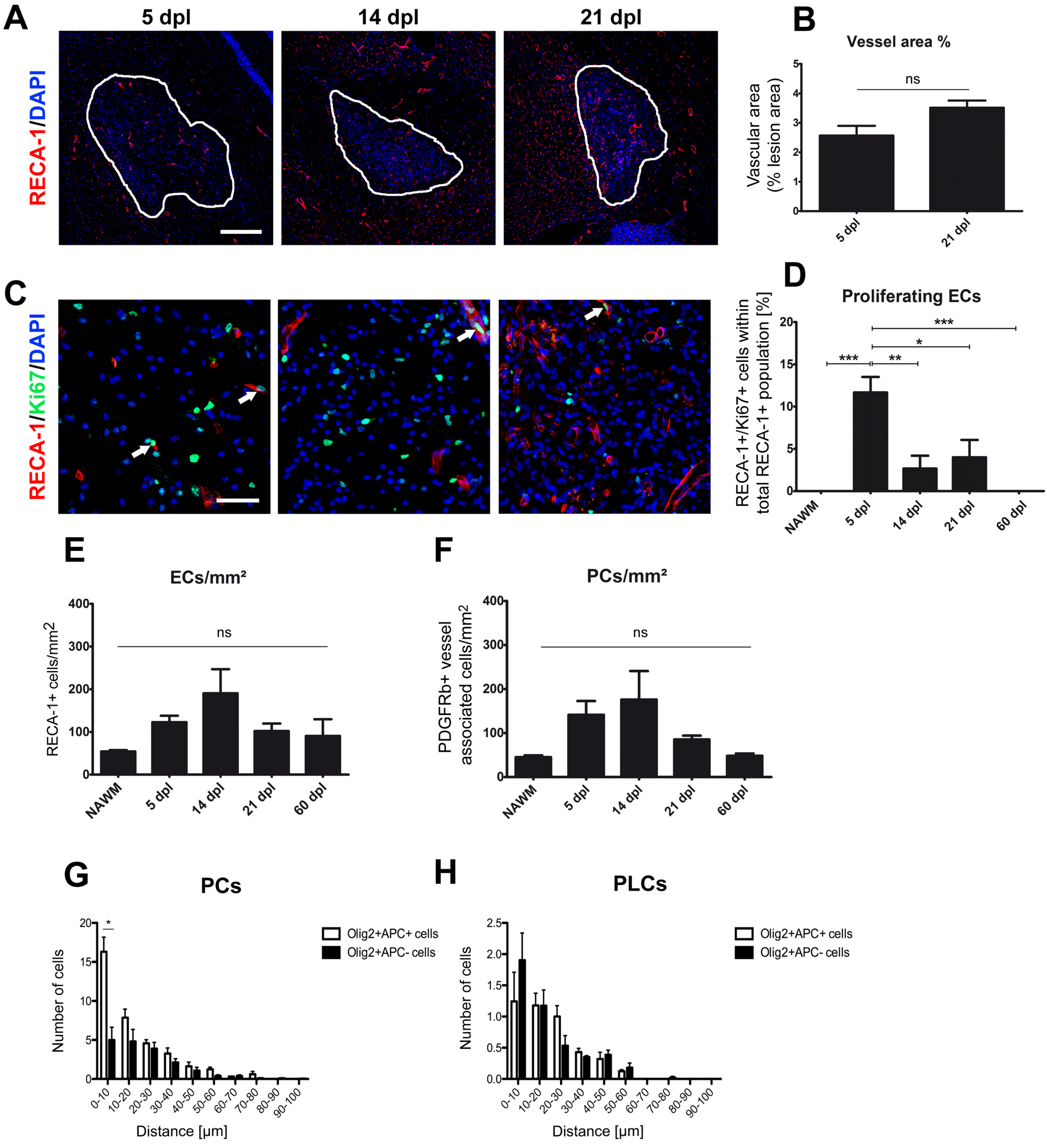
A
5 dpl
14 dpl
21 dpl
RECA-1/DAPI
B
Vessel area %
Vascular area (% lesion area)
ns
5 dpl
21 dpl
C
RECA-1/Ki67/DAPI
D
Proliferating ECs
RECA-1+/Ki67+ cells within total RECA-1+ population [%]
***
**
*
NAWM
5 dpl
14 dpl
21 dpl
60 dpl
E
ECs/mm²
RECA-1+ cells/mm2
ns
F
PCs/mm²
PDGFRb+ vessel associated cells/mm2
G
PCs
Number of cells
Olig2+APC+ cells
Olig2+APC- cells
Distance [µm]
H
PLCs

Figure S1

Figure S1: ECs proliferate during remyelination, Related to Figure 1

(A) Confocal images showing RECA-1 staining with indicated lesion area in ethidium bromide demyelinated rat CCP (5, 14, 21 dpl). (B) Graph displaying fraction of RECA-1+ area within lesion area at 5 and 21 dpl. (C) Confocal images illustrating typical RECA-1/Ki67 staining. Arrows indicate proliferating ECs (RECA-1+/Ki67+). (D) Graph showing proliferating ECs within the total EC population. Graphs display (E) total EC numbers (RECA-1+) and (F) total PC (vessel associated PDGFRb+) numbers within the lesion area, during remyelination. (G, H) Graphs displaying the frequency distribution of Olig2+APC+ and Olig2+ APC- cells and their distance to PCs and PLCs. $n \geq 3$ animals were analyzed for each condition and time point. Quantified data are represented as mean ± SEM. Data were analyzed by one-way ANOVA followed by Tukey's post hoc test, student's T test or Chi square distributions respectively $^{*}p < 0.05$, $^{**}p < 0.01$, $^{***}p < 0.001$. Scale bars: (A, C) 300 μm.

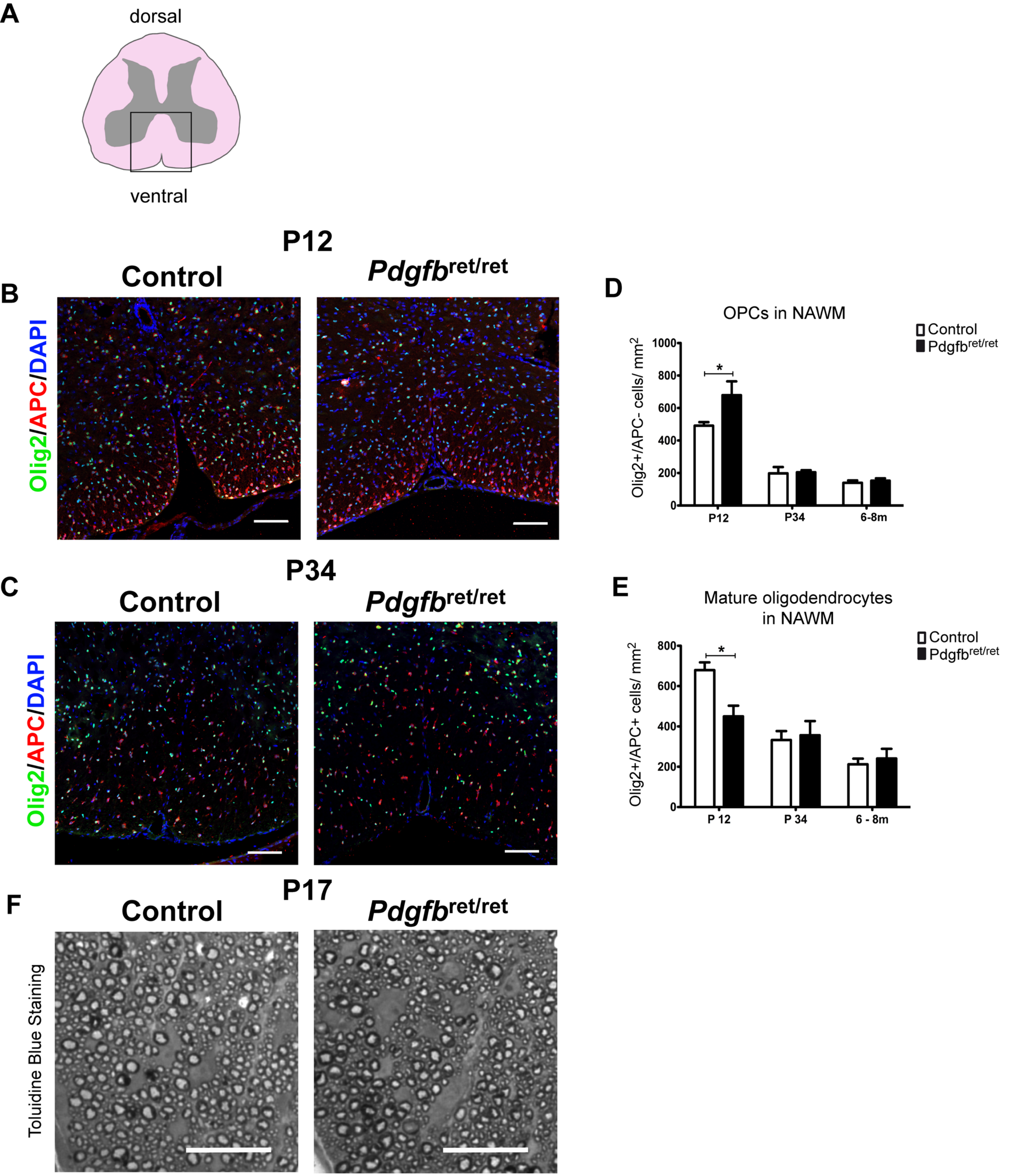
A
dorsal
ventral
P12
B
Control
Pdgfb ret/ret
Olig2/APC/DAPI
D
OPCs in NAWM
Control
Pdgfb ret/ret
Olig2+/APC- cells/ mm2
1000
800
600
400
200
0
*
P12
P34
6-8m
P34
C
Control
Pdgfb ret/ret
Olig2/APC/DAPI
E
Mature oligodendrocytes
in NAWM
Control
Pdgfb ret/ret
Olig2+/APC+ cells/ mm2
800
600
400
200
0
*
P 12
P 34
6 - 8m
P17
F
Control
Pdgfb ret/ret
Toluidine Blue Staining

Figure S2

Figure S2: Oligodendrocyte differentiation is delayed in *Pdgfb*$^{ret/ret}$ mice development, Related to Figure 2

(A) Schematic illustration of the analyzed white matter region in the unlesioned spinal cord. (B, C) Confocal images display mature oligodendrocytes (Olig2+/APC+) in spinal cord ventral NAWM at P12 and P34. (D) Graph shows content of Olig2+/APC- cells / mm^2 and (E) Olig2+/APC+ cells/mm^2 of NAWM in P12, P34 and 6-8 months old control and *Pdgfb*$^{ret/ret}$ mice. (F) Light microscope images display myelination in the ventral spinal cord area at P17 observed by toluidine blue staining. n = 2 animals were analyzed for each condition for immunofluorescence and n=3 animals were analyzed for each condition by toluidine blue staining. Quantified data are presented as means ± SEM. Data were analyzed by two-way ANOVA followed by Bonferroni's post hoc test. *$p<0.05$. Scale bar 100 μm.

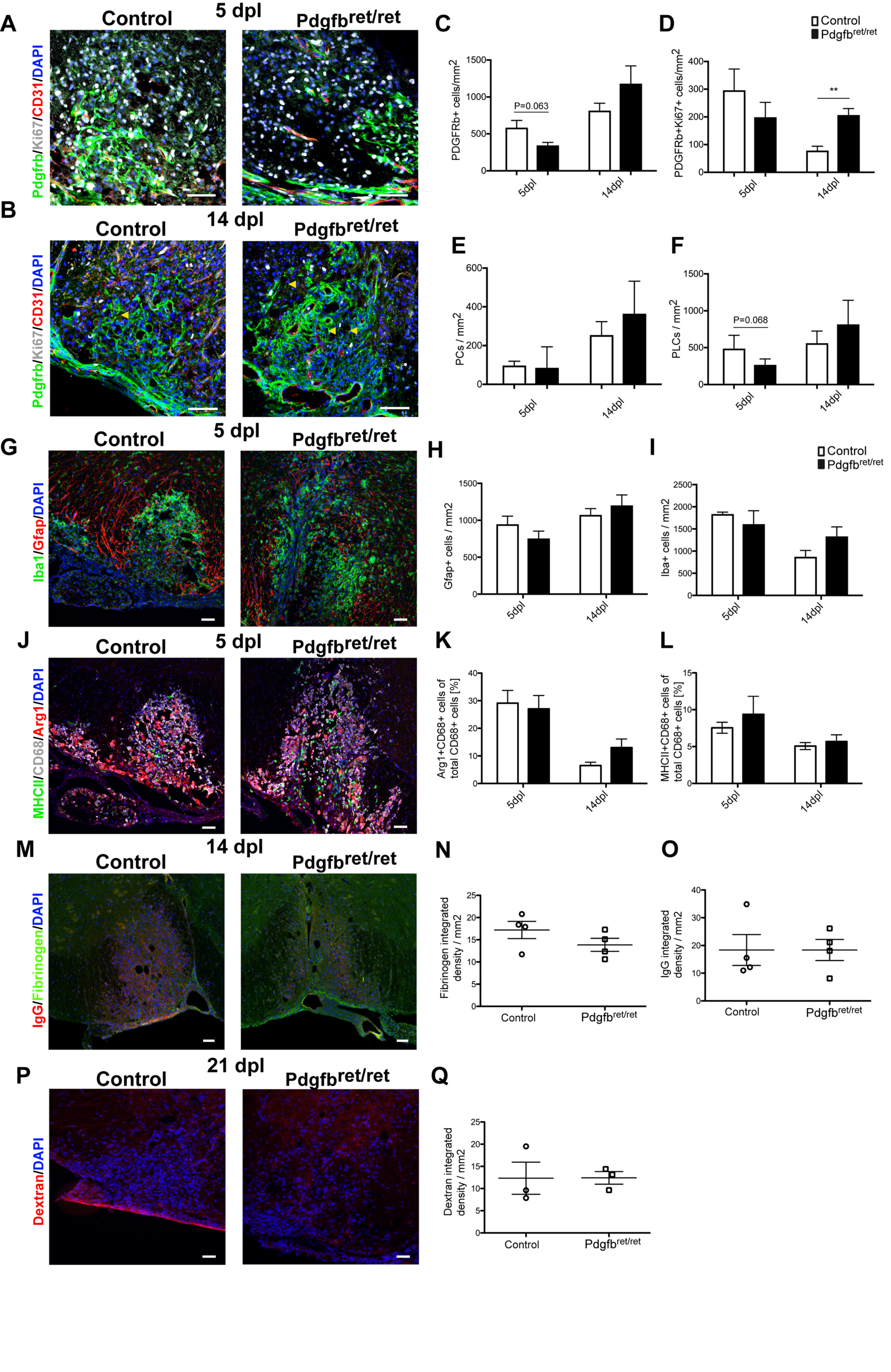

A
B
C
D
E
F
G
H
I
J
K
L
M
N
O
P
Q
Control
Pdgfbret/ret
5 dpl
14 dpl
21 dpl
Pdgfrb/Ki67/CD31/DAPI
Iba1/Gfap/DAPI
MHCII/CD68/Arg1/DAPI
IgG/Fibrinogen/DAPI
Dextran/DAPI
PDGFRb+ cells/mm2
PDGFRb+Ki67+ cells/mm2
PCs / mm2
PLCs / mm2
Gfap+ cells / mm2
Iba+ cells / mm2
Arg1+CD68+ cells of total CD68+ cells [%]
MHCII+CD68+ cells of total CD68+ cells [%]
Fibrinogen integrated density / mm2
IgG integrated density / mm2
Dextran integrated density / mm2
P=0.063
P=0.068
**
5dpl
14dpl

Figure S3

Figure S3: PDGFRb+ cells show a high proliferation rate in *Pdgfb*$^{ret/ret}$ mice upon lysolecithin lesion induction, Related to Figure 2

(A, B) Confocal images show the PC and PLC distribution and proliferation in the lesion at 5 and 14dpl. (C-F) Graphs display the quantification of PDGFRb+ cells and their proliferation. PDGFRb+ cells density is lower at 5dpl but catches up at 14dpl showing a high proliferative response in the *Pdgfb*$^{ret/ret}$ mice. (G-I) Immunostaining and quantification showing astrocyte and microglia/macrophage cell marker expression in the lesion in control and *Pdgfb*$^{ret/ret}$ mice. (J-L) Confocal images and graphs showing the expression of pro-inflammatory and anti-inflammatory microglia/macrophages in the lesion of control and *Pdgfb*$^{ret/ret}$ mice. (M) Immunostaining of fibrinogen and IgG at 14dpl in control and *Pdgfb*$^{ret/ret}$ mice to determine blood brain barrier stability. (O) Graph displays quantification of fibrinogen and IgG integrate density per lesion area. (P) Images showing immunostaining for dextran extravasation at 21dpl in lesions from control and *Pdgfb*$^{ret/ret}$ mice. (Q) Graph displays the quantification on the dextran's integrated density per lesion area in control and *Pdgfb*$^{ret/ret}$ mice. n=4 mice per condition were analyzed at 5 and 14dpl and n=3 mice were analyzed at 21dpl. Quantified data are represented as means ± SEM. Data were analyzed by Students t-test or U-Mann Whitney test per time point. $^{**}p<0.01$. Scale bar 50µm.

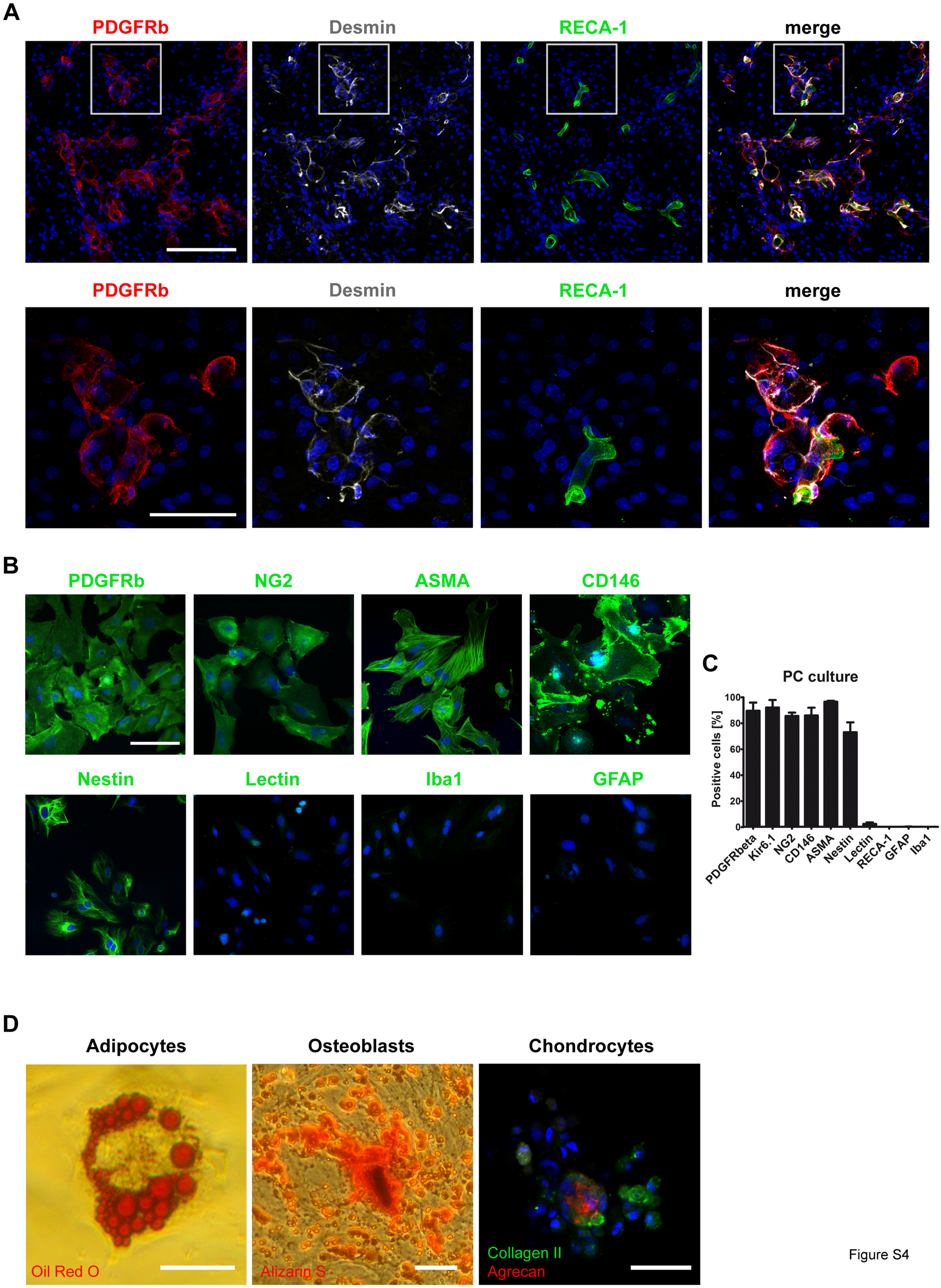
A
PDGFRb
Desmin
RECA-1
merge
PDGFRb
Desmin
RECA-1
merge
B
PDGFRb
NG2
ASMA
CD146
Nestin
Lectin
Iba1
GFAP
C
PC culture
Positive cells [%]
100
80
60
40
20
0
PDGFRbeta
Kir6.1
NG2
CD146
ASMA
Nestin
Lectin
RECA-1
GFAP
Iba1
D
Adipocytes
Osteoblasts
Chondrocytes
Oil Red O
Alizarin S
Collagen II
Agrecan

Figure S4

Figure S4: Immunohistological characterization of PCs, Related to Figure 3

(A) Confocal images showing expression of PDGFRb, desmin and RECA-1 in lesion area of ethidium bromide demyelinated rats (21 dpl). Boxed areas are shown in the lower panel in higher magnification. (B) Fluorescence images display cultured PCs positive for the PC-specific markers PDGFRb, NG2, ASMA, CD146, Nestin, and did not stain with Lectin, a staining indicating EC, the microglial marker Iba1 and the astrocyte marker GFAP. (C) Graph shows quantification of different markers expressed by cultured PCs. (D) Phase contrast and fluorescence images showing mesenchymal lineage differentiation potential of cultured PCs: adipocytes (Oil red O); osteoblasts (Alizarin S) and chondrocytes (Collagen II and Agrecan). Scale bars: (A) 100 µm, high magnification 20 µm, (B) 100 µm, (D) 50 µm.

Supplementary Experimental Procedures

Animals, surgical procedures and tissue processing

Animal experiments within this research have been regulated under the Animals (Scientific Procedures) Act 1986 Amendment Regulations 2012 following ethical review by the University of Cambridge Animal Welfare and Ethical Review Body (AWERB) in accordance with UK Home Office regulations (Project License: 70/7715) and in accordance with Austrian laws on animal experimentation and were approved by Austrian regulatory authorities (Permit No. BMWF-66.012/0001-II/3b/2014; license codes BMBF-66-012/0037-WF/V/3b/2014 and BMWF-66.012/0032-WF/V/3b/2015). During this study, 2-3 months old Sprague Dawley rats and 12 weeks old *Pdgfb*$^{ret/ret}$ mice (hetero- and homozygous), which were previously described in (Armulik et al., 2010; Lindblom et al., 2003; Nystrom et al., 2006)), were used for toxin induced demyelinations. Rats were anesthetized by inhalation of Isoflurane/Oxygen (2-2.5 O_2 1000ml/min) under analgesia (Buprenorphine 0.05mg per kg body weight) and demyelinated in the caudal cerebellar peduncles by ethidium bromide local injection as previously described in (Woodruff and Franklin, 1999). Rats were sacrificed at different time points 5, 14, 21 and 60 dpl by transcardial infusion of 4% paraformaldehyde (PFA) (under terminal anaesthesia) and brains were post-fixed overnight in 4% PFA (at 4 °C). Tissue was cut on a cryostat in 12 µm slices.

Mice were anesthetized with 65 mg/kg Ketamin, 13 mg/kg Xylazin and 2 mg/kg Acepromacin by intra peritoneal injection or by inhalation of Isoflurane/Oxygen (2-2.5 O_2 1000ml/min) under analgesia (Buprenorphine). Local Lysolecithin-driven demyelination in mice was induced in the ventrolateral spinal white matter as previously described in (Fancy et al., 2009). Mice were sacrificed 5dpl, 14 dpl and 21dpl by transcardial infusion of 4% PFA under terminal anesthesia and spinal cord tissue was post-fixed for 6 hrs in 4% PFA (at 4°C). Tissue was cut on a cryostat in 12 µm slices. For toluidine blue experiments mice were sacrificed at 21dpl by transcardial infusion of 4% Glutaraldehyde under terminal anesthesia and spinal cord tissue was post-fixed for 2h in 4% Glutaraldehyde (at 4°C). Tissue was embedded in resin and cut in 1µm resin sections with the microtome.

For the blood brain barrier stability experiment (Dextran extravasation measurement) 10kDA biotin conjugated Dextran molecule (Sigma Aldrich) was injected intravenously to each mice and 15min after the mice were euthanized without intracardial perfusion. Spinal cords were post- fixed in 4% PFA and then embedded in OCT for further cryostat sectioning.

Transgenic mice

PDGF-B retention motif knock-out (*Pdgfb*$^{ret/ret}$, pericyte-deficient) and control (heterozygous littermates) mice (Lindblom et al., 2003) were used in this study either alone or crossed with Pdgfrb-eGFP mice (Gensat.org. Line name: Tg(PdgfrbeGFP)JN169Gsat/Mmucd). Pericyte-deficient mice have only 15-25% of brain capillaries covered by pericytes as compared to the control mice (Armulik et al., 2010). Animal experiments were approved by the Stockholm North Ethical Committee on Animal Research (Permit number N16/12), the Uppsala Ethical Committee on Animal Research (Permit number: C224/12 and C225/12), and by the Cantonal Veterinary Office Zurich, Switzerland (ZH196/2014).

Preparation of primary CNS pericytes

Preparation was performed following Dore-Duffy protocol (Dore-Duffy et al., 2006) with modifications. Briefly, rat primary pericytes (PCs) were isolated from 6 – 8 weeks old female Fisher 344 rats. 5 Rats were anesthetized by Isofluran and subsequently decapitated. Brains were collected in ice-cold alphaMEM (Gibco). Meninges were removed properly and brains were minced in alphaMEM by a douncer until homogenized. Half of a centrifugation tube was filled with 4°C cold 15% dextran solution in alphaMEM (autoclaved) and carefully overlayed with brain homogenate and centrifuged at 5000 g at 4°C for 10 min. Pellet containing cerebral vessels was collected and washed once with alphaMEM (1000 g, 10 min, 4°C). Vessels were resuspended in alphaMEM and filtered through a 150µm mesh and the flow-through was again filtered through a 40 µm mesh to get rid of single cells. Microvessels were collected in alphaMEM. After centrifugation (1000g, 10 min, 4°C), microvessels were digested in a waterbath for 30 min at 37°C

with 0.01% papain (Worthington Biochemicals, England), 0.1% dispase II (Sigma), 0.01% DNase I (Worthington Biochemicals), 0.1% collagenase 1 (Life Technologies) and 12.4 mM $MgSO4$ in HBSS without $Mg2+/Ca2+$ (Life Technologies). Every 10min digested microvessels were resuspended and centrifuged (1000 g, 10 min) and the pellet collected. Cells were cultured in 1 well of a 12 well plate in alphaMEM containing 20% FBS (Gibco) and 100U/ml Penicillin/ 100µg/ml Streptomycin (Thermo Fisher) in a humidifying incubator (20% O_2, 5% CO_2 at 37°C). PCs were cultured in alphaMEM containing 20% FBS and from passage 1 they were cultured in alphaMEM containing 10% FBS. Passaging was done by 0.25% Trypsin in PBS. Passages 6-10 were used for the experiments.

Preparation of primary bone marrow-derived mesenchymal stem cells

Rat bone marrow-derived mesenchymal stem cells (MSCs) were prepared as previously described in (Rivera et al., 2006). Briefly, 6 – 8 weeks old female Fisher 344 rats were anesthetized by Isofluran and sacrificed. Bones were isolated from the hind limbs and bone marrow was washed out and dissociated in a petri dish using a filled syringe with alphaMEM. Bone marrow was washed twice with alphaMEM and seeded in a P100 petri dish in alphaMEM containing 100U/ml Penicillin/ 100µg/ml Streptomycin (Thermo Fisher) and 10% FBS and cultured in a humidifying incubator (20% O_2, 5% CO_2 at 37°C).

Preparation of Oligodendrocyte Progenitor Cells

OPCs were prepared from neonatal P0-2 Sprague Dawley rats. Rats were anesthetized with Pentobarbital and sacrificed. After removal of the hindbrain/midbrain, olfactory bulbs and meninges, cortices and hippocampi were digested with papain solution (1.2U/mL cysteine protease papain (Worthington), 3.7mg/mL DNase I type IV, 2mM L-Cysteine and 1U/mL Penicillin/ Streptomycin (Sigma Aldrich) in DMEM (Gibco). Dissociated cells were seeded in 5µg/mL poly-D-lysine coated T75 flasks. Mixed glial cultures were kept for 11DIV in medium with DMEM (Gibco), 10% fetal bovine serum (Biosera) and Mycozap PR-Plus (Lonza) prior to OPC isolation. OPCs were isolated as previously described (McCarthy and de Vellis, 1980). OPCs were seeded on 13mm coverslips in a density of 30000 cells/cm^2 and cultured at 37°C in 5% CO_2 in SATO medium (DMEM (Gibco), 1U/mL Penicillin/Streptomycin, 10 µg/mL BSA, 0.06 µg/mL progesterone, 16.1µg/mL putrescine, 0.005 µg/ mL sodium selenite, 5 µg/mL insulin and 50 µg/mL holo-transferrin (Sigma)). 10ng/mL of PDGF-AA and 10ng/mL bFGF (Preprotech) were added daily to keep cells as precursors. After 3 DIV medium was changed to PC-CM or MSC-CM. Conditioned media was replaced every 2 days and OPCs were fixed with 4% PFA at 0, 2, 4 and 6 days.

Collection of conditioned media

PCs and MSCs were each seeded at a cell density of 12,000 cells per cm^2 and incubated in the appropriate medium. After 3 days of incubation, medium was filtered using a 0.22 µm filter and kept at 4°C until use. Independent cell preparations were used for each conditioned media collection and each experiment was done with different conditioned media.

Differentiation of pericytes

For differentiation into adipocytes, PCs were seeded in 12 well plates and treated with AdipoLife^{TM} DfKt-1TM Adipogenesis Medium (Lifeline Cell technology) for 2 weeks. For differentiation into osteoblasts, PCs were seeded in 6 well plates and treated with OsteoLife^{TM} (Lifeline Cell technology) osteogenesis medium for 3 weeks. For chondrocyte differentiation, PCs were pelleted in 15 ml Falcon tubes and treated with ChondroLife™ chondrocyte differentiation medium (Lifeline Cell technology) for 3 weeks.

Blocking of Lama2 in pericyte-derived conditioned medium

PC-CM was collected as described above. To block Lama2 in the PC-CM, the medium was incubated with a 1:50 dilution of either general rabbit IgG antibody (Santa Cruz Biotechnologies) or rabbit anti-Lama2 antibody (Santa Cruz Biotechnologies) in rotation for 2h at room temperature and covered from light. Following incubation, the OPC medium was replaced by the PC-CM (pre-incubated with the different antibodies). This process was repeated with every medium change (at 0, 2 and 4 DIV). As a control for the effect of the antibodies in OPCs, the pre- incubation with the corresponding antibodies was also performed for the control unconditioned medium in parallel.

PCs were trypsinized from 10cm dishes, counted and seeded at a density of 75000 cells per 12 well. The following day media was changed to basic OPC control media without P/S and the next morning PCs were transfected using Lipofectamine siRNA max. PCs were transfected using Opti-Mem (Gibco), 2.5µl Lipofectmine RNAi max (Invitrogen) and 50nM of non-targeting or rat –Lama2 siRNA (Dharmacon) per 12 well. After 6h media with transfections reagents was removed and fresh OPC control media added. OPC control media was incubate for 48h with the transfected PCs and then collected and filtered through a 0.22µm filter prior to its addition to OPCs.

Demyelination of organotypic slices

Remyelination of rat cerebellar slices was prepared as previously described (Birgbauer et al., 2004). Briefly, P9 Sprague Dawley rats were euthanized by overdose of anesthetic and the cerebellum was cut in 300 µm thick sagittal slices using a McIllwain Tissue chopper. Slices were incubated on 4.0µm pore 30mm diameter filter (Millipore) for 7 DIV in organotypic slice medium (50% basal Eagle medium (BME) (Gibco), 25% heat inactivated horse serum (Gibco), 25% HBSS (BME) (Gibco), 5mg/mL glucose, 1X Glutamax (Gibco) and 1X Mycozap – PR plus (Lonza)), with medium changes every other day. After 7 DIV, medium was replaced by organotypic slice medium containing 0.5mg/mL lysolecithin for 16h. After 16h medium was replaced by PC conditioned organotypic slice medium and non-conditioned control medium. Medium was replaced by new PC-CM every other day and slices were fixed at 0dpl, 2dpl, 4dpl, 6dpl and 8dpl with 4% PFA for 1h at room temperature. Slices stored at -20°C in PBS until immunostaining was performed. Independent slice culture preparations were used to generate the data.

Immunocytochemistry

Pericytes

PCs and MSCs were detached by 0.25% Trypsin and seeded on uncoated glass coverslips at a density of 1×10^4 cells/cm^2. Cells were fixed with 4% paraformaldehyde (PFA) with pH 7.4 for 10 - 15 min and washed 3x with phosphate buffered saline (PBS). Cells were blocked with fish skin gelatin buffer (FSGB) containing TBS (0.15 M NaCl, 0.1 M Tris-HCl, pH 7.5), 0.1% Triton-X100 (only for intracellular antigens), 1% bovine serum albumine (BSA) and 0.2% Teleostean gelatin (Sigma, Germany) for 2hrs at room temperature (RT). Primary antibodies were applied in blocking solution overnight at 4°C. Cells were incubated with fluorochrome-conjugated species-specific secondary antibodies at RT for 2hrs and washed 3x with PBS. To analyze the marker expression profile the following antibodies were used. Primary antibodies: rabbit anti-glial fibrillary acidic protein (GFAP) 1:1000 (Dako); mouse anti-rat Nestin 1:500 (BD Pharmingen); rabbit anti-NG2 1:200 (Millipore); goat anti platelet-derived growth factor receptor beta (PDGFRb) 1:100 (R & D Systems); rabbit anti Kir 6.1 1:100 (Abcam); mouse anti CD146 (MUC18) 1:100 (Invitrogen); mouse anti alpha smooth muscle actin (ASMA) 1:500 (Sigma); rabbit anti Iba1 1:300 (Wako); biotinylated Lectin from *B. simplicifolia* (1:500, Sigma-Aldrich); mouse anti RECA-1 1:500 (Abcam); rabbit anti Desmin 1:200 (Abcam). Secondary antibodies: donkey anti-rabbit conjugated with Alexa 568 1:1000 (Invitrogen); donkey anti-rabbit conjugated with Alexa 488 1:1000 (Invitrogen); donkey anti-rabbit conjugated with Alexa 647 1:1000 (Millipore); donkey anti-goat conjugated with Alexa 488 1:1000 (Invitrogen); donkey anti-goat conjugated with Alex 568 1:100 (Invitrogen); donkey anti-mouse conjugated with Alexa 488 1:1000 (Invitrogen); donkey anti-mouse

conjugated with Alexa 568 1:1000 (Invitrogen); donkey anti-mouse conjugated with Cy5 1:1000 (Jackson); Streptavidin conjugated with Alexa 488 1:500 (Invitrogen); Streptavidin conjugated with Alexa 568 1:500 (Invitrogen, Austria). For the detergent-sensitive antigens (i.e. NG2) Triton X- 100 was omitted from FSGB. Nuclear counterstaining was performed with 4′, 6′-diamidino-2-phenylindole dihydrochloride hydrate 0.25 µg/µl (DAPI; Sigma). Specimens were mounted on microscope slides using Prolong Antifade kit (Molecular Probes).

Oligodendroglial progenitor cells

OPCs were fixed in 4% PFA (pH 7.4) for 10 – 15min and washed twice with PBS for 10 min. Cells were blocked with 5% normal donkey serum (NDS) (Sigma Aldrich) and 0.1% Triton for 1 h at RT. Primary antibodies were applied in 5% NDS and 0.1% Triton over night at 4 °C. Cells were washed and incubated in 5% NDS with fluorochrome-conjugated species-specific secondary antibodies at RT for 2hrs and washed 3x with PBS. To analyze the marker expression profile the following antibodies were used. Primary antibodies: goat anti-Olig2 1:200 (R and D Systems), rabbit anti- Olig2 1:500 (Millipore), mouse anti-2`, 3`-cyclic-nucleotide-3`- phosphodieesterase (CNP) 1:400 (Abcam), rat anti-myelin basic protein (MBP) 1:500 (Serotec), mouse anti- NG2 1:200 (Millipore), rabbit anti-Ki67 1:500 (Abcam) and rabbit anti-active caspase 3, 1:500 (Millipore). Secondary antibodies: donkey anti-rabbit conjugated with alexa 488; donkey anti-mouse conjugated with alexa 647; donkey anti-goat conjugated with alexa 488 and donkey anti-rat conjugated with alexa 568. All secondary antibodies were diluted 1:500 and they were purchased from Invitrogen. Specimens were mounted on microscope slides using fluoromount G (Southern Biotech).

Immunohistochemistry

Cryo sections

For Figures 1, 2, S1, S2, S3 and S4: Immunohistochemistry was performed as previously described in Kazanis et al 2015 (Kazanis et al., 2015). Briefly, cryo sections were warmed to room temperature and rehydrated in PBS. Antigen retrieval was performed with boiled citrate buffer (Sigma) for 10 min and washed 3x 10 min with PBS. Sections were blocked with 10% normal goat or donkey serum and 0.1% triton and incubated with antibodies in appropriate dilutions, washed and mounted with Fluoromount G (Southern Biotech). Primary antibodies: goat anti platelet-derived growth factor receptor beta (PDGFRb) 1:100 (R & D Systems); rabbit anti Desmin 1:200 (Abcam); mouse anti RECA-1 1:500 (Abcam); rabbit anti Olig2 1:300 (Millipore); Mouse anti CC1 1:500 (Calbiochem); rabbit anti Ki67 1:100 (Thermo Scientific); rabbit Ki67 (Abcam), rat anti CD31 (BD Pharmingen); rabbit anti Laminin 1:300 (Sigma Aldrich); rabbit anti CD68 1:500 (Abcam), rat anti MHCII 1:100 (eBiosciences); goat anti Arginase I 1:100 (Santa Cruz Biotechnologies); goat anti Iba1 1:500 (Abcam); chicken anti GFAP 1:1000 (Abcam) and sheep anti fibrinogen 1:200 (Bio-Rad). Stainings of APC on mouse tissue was performed using Mouse On Mouse basic kit (Vector labs). Secondary antibodies: donkey anti-rabbit conjugated with Alexa 568 1:1000 (Invitrogen); donkey anti-rabbit conjugated with Alexa 488 1:1000 (Invitrogen); donkey anti-rabbit conjugated with Alexa 647 1:1000 (Millipore); donkey anti-goat conjugated with Alexa 488 1:1000 (Invitrogen); donkey anti-goat conjugated with Alex 568 1:100 (Invitrogen); donkey anti-mouse conjugated with Alexa 488 1:1000 (Invitrogen); donkey anti-mouse conjugated with Alexa 568 1:1000 (Invitrogen); donkey anti-mouse conjugated with Cy5 1:1000 (Jackson); donkey anti-chicken Alexa 594 (Invitrogen), Streptavidin conjugated with Alexa 488 1:500 (Invitrogen); Streptavidin conjugated with Alexa 568 1:500 (Invitrogen, Austria). For APC stainings, donkey anti Mouse IgG2b Alexa 647 or Alexa 488 was used. Nuclear counterstaining was performed with Hoechst (Sigma).

For Figure 4: immunohistochemistry was carried out on vibratome cut sagittal sections (50µm). Tissues were blocked and permeabilized in PBS containing 2.5% donkey serum, 1% bovine serum albumin and 0.5% TritonX-100 in PBS overnight at 4 °C. Tissues were then incubated in primary antibody solution for 48 hours at 4 °C, washed, and subsequently incubated in secondary antibody solution over night at 4 °C. Sections were mounted in Prolong Gold Antifade (Life Technologies). The following primary antibodies were used: goat anti- mouse CD31 (R&D Systems); rabbit anti-desmin (Abcam); rat anti-laminin alpha2 (Abcam). The fluorescently labeled secondary antibodies made in donkey were purchased from Jackson Immunoresearch.

Organotypic Slices

For immunostaining, slices were blocked for 1h with 10% normal goat serum 0.5% Triton X-100 in agitation and incubated overnight with primary antibodies in blocking solution. Slices were washed three times with PBS 0.1% Triton X-100 and then incubated with secondary antibodies for 2h at room temperature. The secondary antibodies were removed washing with PBS 0.1% Triton X-100 and then Hoechst (1µg/mL final concentration, Sigma) was used for nuclei staining. Slices were mounted with Fluoromount G onto poly D- lysine slides and covered with a 20mm coverslip (VWR). Primary antibodies: rat anti-myelin basic protein (MBP) 1:500 (Serotec) and rabbit anti- neurofilament H 1:500, (Abcam). Secondary antibodies: goat anti-rabbit conjugated with Alexa 488, 1:500, (Invitrogen) and goat anti-rat conjugated with Alexa 568 1:500 (Invitrogen).

Developmental myelination and remyelination assessment

Microtome cut 21dpl 1µm slices were stained in a heat plate with Toluidine Blue and the excess of toluidine blue was washed out with MiliQ water and images were taken with light microscopy. For remyelination the different lesions were blindly ranked. Two independent people organized and ranked them according to their level of demyelination and remyelination.

In situ hybridization

The expression of a number of marker mRNA species in demyelinated lesions was examined by *in situ* hybridization with digoxigenin-labelled cRNA probe for *Plp.* Animals were perfused with 4% PFA via the left heart ventricle. The tissue was extracted, post-fixed in 4% PFA overnight at 4ºC and cryoprotected in 20% sucrose. *In situ* hybridizations were conducted on cryostat sections (12 µm) using previously established protocols (Kotter et al., 2006; Zhao et al., 2008).

Fluorescence *in situ* hybridization

In situ RNA hybridization was performed using RNAscope technology (Advanced Cell Diagnostics) following the manufacturer's protocol with minor modifications. Briefly, fresh-frozen brains from 2-month old mice were cut into 16 µm sagittal sections and mounted on SuperFrost Plus glass slides. After dehydration, slides were subjected to RNAscope Multiplex Fluorescent Assay. First, slides were incubated in Pretreat 4 for 20 min at RT. After that, RNAscope probes were hybridized for 2 h at 40 °C and the remainder of the assay protocol was implemented. The fluorescent signal emanating from RNA probes was visualized and captured using a Leica TCS SP8 confocal microscope (Leica Microsystems). Images are presented as 2D maximum intensity projections of ~3 µm z-stacks. According to the Advanced Cell Diagnostics, each mRNA molecule hybridized to a probe appears as a separate small fluorescent dot.

Lama2 mRNA expression by Affymetrix and RNAseq

The Affymetrix array data were downloaded from NCBI GEO database (accession number: GSE15892) published by Armulik et al. (Armulik et al., 2010). The raw Affymetrix data were normalized using Bioconductor GCRMA package (Bioconductor gcrma package, version 2.32.0), and Significance Analysis of Microarrays was performed to identify the significantly differentially expressed genes (Bioconductor Siggenes package, version 1.34.0). Also RNAseq data of Lama2 expression in sorted pericytes vs. microvascular fragments was extracted from our previous publication (He et al., 2016).

Western Blot

Proteins were isolated from rat cerebellum in liquid nitrogen frozen, dissociated with a mortar and resuspended in RIPA buffer. *In vitro* cultured PCs were harvested with a cell scraper and resuspended in RIPA. Tissue and cells were homogenized and after 30 min incubation at 4 °C, lysate was spun at 13000 rpm for 15 min, protein concentration of supernatant was measured using Pierce BCA Protein Assay Kit (Thermo Fisher). 3x 4ml PC- CM and control medium was filtered through a 5kDa Amicon Ultra centrifugal filter unit (4000g, 4°C in a swinging rotor). Western blot was performed using Bio-Rad Mini-PROTEAN® TGX Stain-Free™ Precast gradient gels in a Bio-Rad wet chamber. 10 µg of protein sample and total amount of filtrate was loaded after adding Laemmli (95°C 5min) and run with 120 V. Precision Plus Protein™ Dual Color Standard (Bio-Rad) as well as SeeBlue® Plus2 Pre-stained Protein Standard (Thermo Fisher). Blotting was performed using a Trans- Blot® Turbo™ Transfer System (Bio-Rad) with Trans-Blot® Turbo™ Mini PVDF Transfer Packs (Bio-Rad). Membranes were blocked with 5% BSA in tris buffered saline with 0.1% Triton (TBST) for 1 h at room temperature following primary antibody incubation over night at room temperature (rabbit anti Lama2 1:200 (Santa Cruz); mouse anti beta Actin 1:2000 (SIGMA), washed with TBST and incubated with secondary antibodies in blocking solution for 2 hrs at room temperature (donkey anti rabbit Alexa 488 1:1000 (Invitrogen); donkey anti mouse Alexa 568 1:1000 (Invitrogen)). Antibody-labeled proteins were visualized using Chemidoc (Bio-Rad).

Histological analyses

As all caudal cerebellar peduncle (CCP) lesions have been performed in both hemispheres, analyses were carried out in both lesions and rostral, central and caudal regions were taken into consideration to determine a mean value (cell quantification within lesion area, 2 lesions per animal and different layers of lesion) per animal. Similarly, mean values were determined for ventral spinal cord WM lesions. All cellular quantifications were performed within a defined NAWM (CCP/ ventral spinal cord WM) region or within a lesion area in the respective WM. Lesions were recognized by hypercellularity indicated by DAPI signal. Lesions affecting the neighboring grey matter were excluded from analyses.

Lysolecithin lesions were recognized by its hypercellularity indicated by DAPI signal and images containing the full lesion of at least three section of each animal were acquired by microscopy using Z-Stack. For quantification of the different cells a maximum projection of the confocal stacks was created using Fiji and the different cell types quantified either manually using Cell Counter or using colocalization masks macros in Fiji.

For immunocytochemistry at least 5 fields selected by DAPI were imaged per condition using confocal microscopy stacks. Then using Fiji a maximum projection of each the confocal stacks was created and the different cell types quantified either manually using Cell Counter or using colocalization masks macros in Fiji.

Quantification of PCs and ECs

For quantifications of PCs and ECs, immunohistochemistry against PDGFRb and RECA-1, respectively, counterstained with DAPI was carried out, followed by confocal imaging (z-stacks of 12µm slices and 40x magnification) and analyzed using ImageJ. Only vessel-associated cells displaying processes extending along microvessels, a clear roundish nucleus and PDGFRb expression have been considered as PCs. ECs have been quantified following criteria such as a clear RECA-1 staining and a clearly visible cigar-shaped or elongated nucleus (within the microvasculature). A PDGFRb or RECA-1 staining without nucleus (or without a clearly identifiable round/cigar-shaped or elongated nucleus) has not been taken into consideration. Quantification of PLCs followed the same basic criteria, such as a clear PDGFRb staining as well as clear round nucleus. These cells in turn, were counted as PLCs only if their processes were completely detached from vessels. PLCs were not considered when a RECA-1 signal (even if without nucleus) was found in close vicinity. Following these criteria the EC:PC ratio was determined upon quantification of EC and PC total numbers within the same area.

Cell distance measurements

We analyzed the CCP / ventral spinal cord WM lesions at 14dpl. To evaluate the distance between Olig2+ cells and RECA-1+ or PDGFRb+ cells, we used Z-stack confocal images taken of demyelination lesions (12µm slices), as described above. Measurements were performed using ImageJ distance tool. Only PCs and ECs with visible nucleus and clear staining were considered. Only the shortest distance starting from each Olig2-expressing nucleus present within the lesion to the edge of the respective closest RECA-1 or PDGFRb signal was determined. Similarly, as described above, mean values have been determined per animal.

To analyze maturation of oligodendrocytes (Olig2 and APC) and its proximity to PDGFRb+ cells, we performed PDGFRb/Olig2/APC/RECA1 stainings. Distance from an Olig2+ cells in straight line to the center of the closest PDGFRb+ cells was measured. Then both cells were analyzed to observed if the cells were APC+ or APC- and if the PCs were attached to blood vessels (PCs) or not (PLCs). Based on these criteria, we created a plot with the averaged frequency within the three animals and created a frequency distribution for Olig2+ APC- and Olig2+ APC+ cells.

Statistical analyses

Graphs show mean values ± SEM and statistical analysis have been performed using GraphPad Prism 5.0 (GraphPad Software Inc.) and SPSS 20 (IBM). P values below 0.05 were considered to be significant after parametric one-way ANOVA- Tukey post hoc analyses, Student t-Test or U-Mann Whitney tests (when not normally distributed). For statistical analysis with two parameters, such as time course experiments with different treatments, two-way ANOVA-Bonferroni post hoc was used. In the case of positive interaction between the two parameters, as it masks the potential differences between groups, the time parameter was separated and each of the time points analyzed individually as a two-group one parameter using either Student t-test or U-Mann Whitney. For statistical analyses comparing one parameter between only two groups, two-tailed Student's t test or U-Mann Whitney has been performed. For distance frequency distribution analysis Chi square test was used (Figure1M and S1G, H). All experiments were performed the n number indicated in the figure legend. Significance was *<0.05 **<0.01 and ***<0.001.

Supplemental References

Armulik, A., Genove, G., Mae, M., Nisancioglu, M.H., Wallgard, E., Niaudet, C., He, L., Norlin, J., Lindblom, P., Strittmatter, K., et al. (2010). Pericytes regulate the blood-brain barrier. Nature 468, 557-561.

Birgbauer, E., Rao, T.S., and Webb, M. (2004). Lysolecithin induces demyelination in vitro in a cerebellar slice culture system. Journal of Neuroscience Research 78, 157-166.

Dore-Duffy, P., Katychev, A., Wang, X., and Van Buren, E. (2006). CNS microvascular pericytes exhibit multipotential stem cell activity. Journal of cerebral blood flow and metabolism: official journal of the International Society of Cerebral Blood Flow and Metabolism *26*, 613-624.

Fancy, S.P., Baranzini, S.E., Zhao, C., Yuk, D.I., Irvine, K.A., Kaing, S., Sanai, N., Franklin, R.J., and Rowitch, D.H. (2009). Dysregulation of the Wnt pathway inhibits timely myelination and remyelination in the mammalian CNS. Genes & development *23*, 1571-1585.

He, L., Vanlandewijck, M., Raschperger, E., Andaloussi Mae, M., Jung, B., Lebouvier, T., Ando, K., Hofmann, J., Keller, A., and Betsholtz, C. (2016). Analysis of the brain mural cell transcriptome. Scientific reports *6*, 35108.

Kazanis, I., Feichtner, M., Lange, S., Rotheneichner, P., Hainzl, S., Oller, M., Schallmoser, K., Rohde, E., Reitsamer, H.A., Couillard-Despres, S., *et al.* (2015). Lesion-Induced Accumulation of Platelets Promotes Survival of Adult Neural Stem / Progenitor Cells. Experimental neurology *269*, 75-89.

Kotter, M.R., Li, W.W., Zhao, C., and Franklin, R.J. (2006). Myelin impairs CNS remyelination by inhibiting oligodendrocyte precursor cell differentiation. The Journal of neuroscience: the official journal of the Society for Neuroscience *26*, 328-332.

Lindblom, P., Gerhardt, H., Liebner, S., Abramsson, A., Enge, M., Hellstrom, M., Backstrom, G., Fredriksson, S., Landegren, U., Nystrom, H.C., et al. (2003). Endothelial PDGF-B retention is required for proper investment of pericytes in the microvessel wall. Genes & Development 17, 1835-1840.

McCarthy, K.D., and de Vellis, J. (1980). Preparation of separate astroglial and oligodendroglial cell cultures from rat cerebral tissue. The Journal of cell biology *85*, 890-902.

Nystrom, H.C., Lindblom, P., Wickman, A., Andersson, I., Norlin, J., Faldt, J., Lindahl, P., Skott, O., Bjarnegard, M., Fitzgerald, S.M., et al. (2006). Platelet-derived growth factor B retention is essential for development of normal structure and function of conduit vessels and capillaries. Cardiovascular research 71, 557-565.

Rivera, F.J., Couillard-Despres, S., Pedre, X., Ploetz, S., Caioni, M., Lois, C., Bogdahn, U., and Aigner, L. (2006). Mesenchymal stem cells instruct oligodendrogenic fate decision on adult neural stem cells. Stem Cells 24, 2209-2219.

Woodruff, R.H., and Franklin, R.J. (1999). Demyelination and remyelination of the caudal cerebellar peduncle of adult rats following stereotaxic injections of lysolecithin, ethidium bromide, and complement/anti- galactocerebroside: a comparative study. Glia *25*, 216-228.

Zhao, C., Fancy, S.P., ffrench-Constant, C., and Franklin, R.J. (2008). Osteopontin is extensively expressed by macrophages following CNS demyelination but has a redundant role in remyelination. Neurobiology of disease *31*, 209-217.
